# Simulated brain networks reflecting progression of Parkinson’s disease

**DOI:** 10.1162/netn_a_00406

**Published:** 2024-12-10

**Authors:** Kyesam Jung, Simon B. Eickhoff, Julian Caspers, UKD-PD team, Oleksandr V. Popovych

**Affiliations:** Institute of Neurosciences and Medicine - Brain and Behaviour (INM-7), Research Centre Jülich, 52425 Jülich, Germany; Institute for Systems Neuroscience, Medical Faculty and University Hospital Düsseldorf, Heinrich Heine University Düsseldorf, 40225 Düsseldorf, Germany; Department of Diagnostic and Interventional Radiology, Medical Faculty and University Hospital Düsseldorf, Heinrich Heine University Düsseldorf, 40225 Düsseldorf, Germany

**Keywords:** Parkinson’s disease, Brain network, Whole-brain model, Progressive disease

## Abstract

The neurodegenerative progression of Parkinson’s disease affects brain structure and function and, concomitantly, alters the topological properties of brain networks. The network alteration accompanied by motor impairment and the duration of the disease has not yet been clearly demonstrated in the disease progression. In this study, we aim to resolve this problem with a modeling approach using the reduced Jansen-Rit model applied to large-scale brain networks derived from cross-sectional MRI data. Optimizing whole-brain simulation models allows us to discover brain networks showing unexplored relationships with clinical variables. We observe that the simulated brain networks exhibit significant differences between healthy controls (*n* = 51) and patients with Parkinson’s disease (*n* = 60) and strongly correlate with disease severity and disease duration of the patients. Moreover, the modeling results outperform the empirical brain networks in these clinical measures. Consequently, this study demonstrates that utilizing the simulated brain networks provides an enhanced view of network alterations in the progression of motor impairment and identifies potential biomarkers for clinical indices.

## Author Summary

Understanding the progression of neurodegenerative diseases is of extreme importance in medicine. We utilize biophysical whole-brain models to describe how the brain networks change in Parkinson’s disease (PD). We demonstrate clear correlations between the severity of motor impairment and the properties of the simulated brain networks, which are not prominent in empirical brain networks. Furthermore, we show that healthy participants exhibit a pronounced adaptation of network efficiencies in response to varying parameters of the model, while such an adaptation process is suppressed in PD patients with higher disease severity and duration. Our findings suggest a potential model-based biomarker for classification and clinical evaluation of progressive PD using cross-sectional clinical MRI data.

## INTRODUCTION

Parkinson’s disease (PD) is associated with the degeneration of dopaminergic neurons in the substantia nigra pars compacta (Kalia & Lang, 2015). This dopamine deficiency involving basal ganglia circuits leads to movement disorders (DeLong & Wichmann, 2007). As a neurodegenerative disease, PD progresses over time. Evidently, the disease duration is associated with the severity of motor impairment (Holden et al., 2018). Accordingly, taking care of the symptom severity after disease onset is crucial to the quality of patients’ lives. Medication with levodopa or dopaminergic therapy is an effective treatment for PD without diminished effects over a long disease duration of decades (Gupta et al., 2019; Santos-García et al., 2023). However, such a prolonged neurologic state before and after the diagnosis may cause corresponding irreversible brain network alteration (Ruppert et al., 2020; Steidel et al., 2022) and drug-induced dyskinesia (Espay et al., 2018) or cognitive deficits (Manza et al., 2017). These degenerative alterations are important for understanding the progression of the disease in premotor or prodromal periods (before the disease onset), which is one of the clinical challenges.

Investigating the changes of brain networks with disease development can help in understanding its pathological progress. Eventually, these changes not only impact whole-brain networks but also correspondingly alter their topological properties. The network representation of the human brain, also known as the human connectome (Sporns et al., 2005), has been widely employed in neuroscientific research for understanding neural coding and information transmission via brain circuits (Bullmore & Sporns, 2009). Various network properties can be used to investigate the human connectome (Bassett et al., 2018), and the graph theory provides effective tools to evaluate the properties of such complex networks from large-scale brain connectivity (Rubinov & Sporns, 2010). Many studies have addressed the relationships between network properties and behavior (Sporns, 2014) including diseased states (Crossley et al., 2014; Fornito et al., 2015; Griffa et al., 2013). In other words, the network-based approach provides features reflecting psychological and clinical attributes (Medaglia et al., 2015). In particular, brain networks of PD patients have been shown to be different from those of healthy participants in terms of network integration and segregation (Olde Dubbelink et al., 2014; Zuo et al., 2022). Besides, the network-based approach can also be employed for patient classification (Jung et al., 2023; Pläschke et al., 2017; Rubbert et al., 2019).

Examining the network alterations of *in vivo* human brain is limited to the recorded empirical data, and accessing or changing the hidden brain parameters necessary for a better understanding of brain dynamics might require invasive recordings or surgical interventions that can only be permitted within a well-justified therapeutic context following the current medical guidelines, if possible at all. On the other hand, *in silico* brain networks derived from whole-brain modeling have no such limitations regarding virtual interventions, for instance, virtual corpus callosotomy (Owen, Li, et al., 2013) or virtual brain resections in epilepsy (Jirsa et al., 2023). In this study, we therefore suggest an approach that utilizes whole-brain dynamical models and the resultant simulated brain networks to enhance their relationship with clinical variables. With this, we probe the simulated network properties by varying model parameters using the Jansen-Rit type model and search for optimal values that provide the best simulation model correspondence to research objectives (Jung et al., 2023). This behavioral network-based model fitting is a novel approach to investigate the relationships between simulated network properties and clinical measures. It allows us to explore in silico brain dynamics for study conditions that are not available for the analysis of empirical human data in vivo.

**Figure 1.**
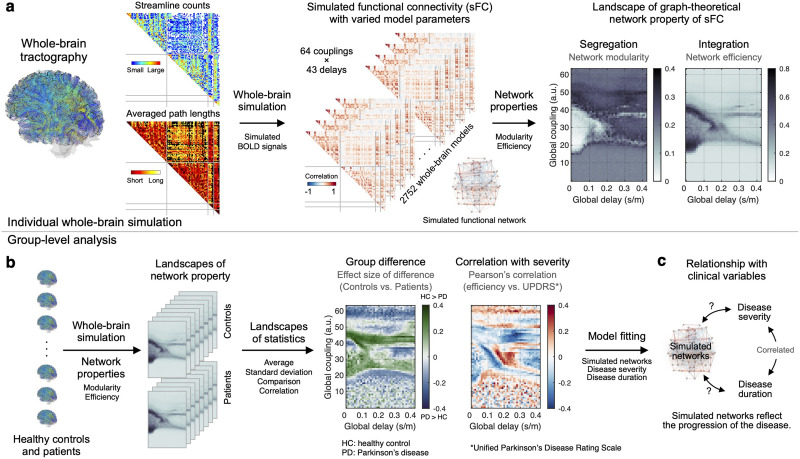
Workflow of the study. (A) Individual WBT calculated from dwMRI data was used to extract parcellation-based empirical SC (streamline counts and streamline path lengths) that was then utilized for derivation of the whole-brain dynamical model, simulation of the resting-state brain activity, and calculation of simulated FC for varying model parameters. For every subject, a few parameter landscapes were obtained, representing the properties of the simulated FC networks, for example, network modularity and efficiency versus model parameters. (B) Individual parameter landscapes of network properties were used for a group-level analysis to obtain a parameter landscape of statistics of simulated network properties across subjects, for example, group differences between patients and healthy controls and correlation between disease severity and network properties. (C) The statistical parameter landscapes and results of the behavioral model fitting were employed for investigation of the relationships between the simulated network properties and clinical variables, for example, disease severity and duration.

Here, we test the relationship between simulated brain networks and clinical scores related to the progression of PD, that are disease severity and disease duration. For the severity, we utilize the Unified Parkinson’s Disease Rating Scale (UPDRS; Goetz et al., 2008) in the modeling approach. It is also used to infer the effect of medication (dopaminergic therapy) on motor impairment with the extent of striatal dopamine depletion (Steidel et al., 2022). Accordingly, we demonstrate an important and intriguing dependence of the modeling results on clinical variables by applying the behavioral network-based model fitting. We specifically opt for network modularity and global efficiency for the whole-brain network segregation and integration, respectively, and address the relationship between the network properties and clinical variables of PD (Figure 1). As a result, we demonstrate significant differences in the simulated network properties between PD patients and healthy controls as well as correlations with disease severity and duration, which were found to be unclear in empirical brain networks. Our results therefore reveal that simulated brain networks clearly reflect the clinical properties of the disease, and the suggested model-fitting approach contributes to investigating and better understanding the disease progression. In consequence, it will possibly provide a new observer-independent biomarker for disease progression.

## RESULTS

### Relationships Between Clinical Variables

We calculated Pearson’s correlation coefficients between clinical and demographic variables. The UPDRS part III (UPDRS III) for motor examination was used in a condition under regular medication (UPDRS III On) and after at least 12 hr of withdrawal of all dopaminergic drugs (UPDRS III Off). In this study, we used UPDRS III On as the disease severity measure for analysis. The severity of motor impairment based on the UPDRS III scores does not significantly correlate with age, disease onset age, and disease duration in the considered cross-sectional data (Table 1). We observed a weak positive correlation between UPDRS III Off (condition without medication) and disease duration. This tendency is consistent with an increase in UPDRS III Off with disease duration in a longitudinal study (Holden et al., 2018). Instead, UPDRS III Off and UPDRS III On (condition with medication) strongly correlate with each other (Supporting Information Figure S1A). As for the effect of the medication, the within-subject difference of UPDRS III (Off − On) shows significant correlations with onset age and the duration of the disease, but this is not the case for age (Table 1, Supporting Information Figure S1B). For a progressive disease, its duration is obviously a more important factor when delving into the effect of medication compared with age.

**Table 1.** Demography and relationship among clinical variables

**Groups**	**Female**	**Male**	**Age**	**Onset age**	**Disease duration**	**UPDRS III Off**	**UPDRS III On**	**UPDRS III Off − UPDRS III On**
Controls	21	30	55.02 (9.78)	N/A	N/A	N/A	N/A	N/A
Patients	17	43	61.95 (9.32)	53.22 (9.30)	8.65 (5.21)	35 (26–40)	17.5 (11–28)	13.5 (7–18)
**Variables (60 patients)**				**Onset age**	**Disease duration**	**UPDRS III Off**	**UPDRS III On**	**UPDRS III Off − UPDRS III On**
Age				0.835**	0.291*	0.024	0.101	−0.109
Onset age				–	−0.284*	−0.080	0.189	−0.366**
Disease duration				–	–	0.180	−0.152	0.446**
UPDRS III Off				–	–	–	0.726**	0.300*
UPDRS III On				–	–	–	–	−0.438**

In the demography, the values of ages (age, onset age, and disease duration) are in years, and the values with the parentheses indicate mean (standard deviation). The values of UPDRS III with the parentheses indicate median (interquartile range). In the relationship, values are Pearson’s correlation coefficients (*n* = 60) denoted by asterisks (*p < 0.05 and **p < 0.005) as significant relationship. Abbreviations: not available (N/A).

### Whole-Brain Model Fitting Using Parameter Landscapes of Network Properties

A neural mass model of the Jansen-Rit type (Jansen & Rit, 1995) was used to simulate excitatory and inhibitory postsynaptic potentials (PSPs) of neural populations in a given brain region (Jung et al., 2023). The brain was parcellated into regions according to the Schaefer atlas with 100 parcels (Schaefer et al., 2018) and the Desikan-Killiany atlas with 68 parcels (Desikan et al., 2006). In addition, we added 14 subcortical areas to each atlas, which were composed into a model network through reconstructed white matter fibers (streamlines), that is the whole-brain tractography (WBT). The number of streamlines connecting two brain regions and their average path length were used to calculate the coupling strength (via the number of streamlines) and delay in coupling (via the path length) between these regions in the model. The excitatory PSPs of the brain regions generated by the model were transformed into the simulated blood oxygenation level-dependent (BOLD) signals by means of the Balloon-Windkessel model (Buxton et al., 1998; Friston et al., 2000). Then, the simulated functional connectivity (FC) was calculated by Pearson’s correlation between the simulated BOLD signals of any pair of the brain regions. This was calculated for many model parameters from a dense grid in the parameter space, where the model parameters were optimized according to a given approach of the model fitting to empirical data. More details can be found in the Methods section.

**Figure 2.**
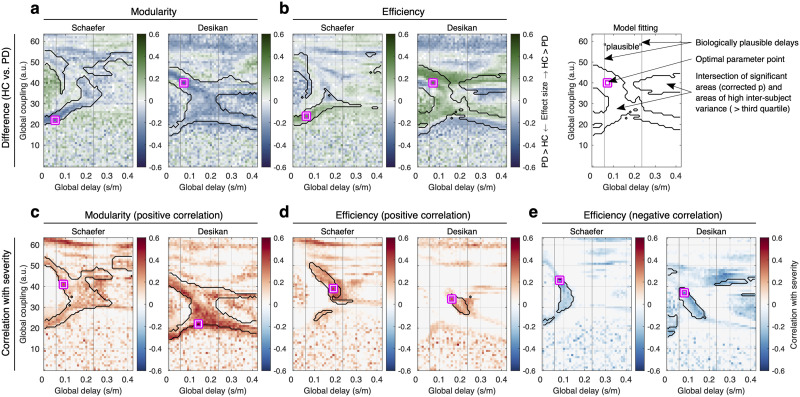
Parameter landscapes of behavioral model fitting of the whole-brain dynamical model of the Jansen-Rit type. The network modularity and network efficiency of simulated FC were used to calculate (A–B) landscapes of the effect size of the group difference between healthy controls (HC) and patients with PD and (C–E) landscapes of Pearson’s correlation (across PD patients) between simulated network properties and severity of the disease, as given by the unified PD rating scales (UPDRS III medication On). The calculations were performed for the Schaefer and the Desikan-Killiany (Desikan) brain atlases indicated in the titles of plots together with the respective network properties. The color depicts the effect size and correlation in plots (A–B) and (C–E), respectively. The vertical lines bound an approximate range of biologically plausible delays; the magenta-white squares indicate the optimal parameter points of the largest effect size or correlation in the parameter domain bounded by the black contour curves of intersection between significant areas thresholded by the random-field theory for multiple tests and areas of high intersubject variance of the respective network properties (> third quartile). See the rightmost plot in the upper row for explanation. Abbreviation: arbitrary unit (a.u.).

In this study, we focused on using two network measures, modularity and global efficiency (Rubinov & Sporns, 2010), to analyze their relationship with clinical measures. We chose these measures because they are well established and interpretable (Deco et al., 2015; Sporns, 2013; Wang et al., 2021) and also related to PD, as previously shown in a meta-analysis (Zuo et al., 2022). Modularity measures network segregation, which is the strength of the community structure of a given network. Global efficiency, on the other hand, is a measure of network integration, which is the average of the inverted shortest path lengths (Rubinov & Sporns, 2010). We calculated the network modularity and efficiency for the weighted graphs of the empirical structural connectivity (SC) and FC as well as simulated FC. In the latter case, the network measures were projected on the two-dimensional model parameter space that comprises the parameters of global coupling and delay (Figure 1A and Methods section). We therefore simultaneously optimized the two global model parameters for a given approach of the model fitting (Methods section), which were frequently utilized for the whole-brain model fitting in many other studies (Deco et al., 2009; Zimmermann et al., 2018). Global coupling was employed to scale the coupling strength in the whole-brain SC used for the underlying model network, while the global delay was utilized to scale the delay in signal propagation through the white matter fibers between brain regions. Each subject, therefore, had landscapes of network modularity and efficiency. By the group-level (across subjects) statistical analyses (Figure 1B), we obtained the respective statistical parameter maps (Figure 2). The latter in particular include parameter regimes showing significant results thresholded by the random-field theory for multiple tests, which preserves significance when spatial correlations are proximate (Ashburner et al., 2003; Worsley et al., 1996). There, parameter domains were overlapped with those of a relatively large (> third quartile) intersubject variability of the network properties for a robust performance against noise; see the Methods section and Supporting Information Figures S2 and S3. The intersections of these regimes were used for the behavioral network-based model fitting as a parameter mask (Supporting Information Figure S2C). Then, we searched for the optimal parameter points (the magenta-white squares in Figure 2) corresponding to the largest effect size of the group difference between healthy controls and patients (Figure 2A–B) and the strongest positive or negative Pearson’s correlation coefficients between the network properties and disease severity (UPDRS III On scores; Figure 2C–E). We in particular found that, in spite of the parameter optimization over the broad parameter range, the optimal global delays were located in the interval [0.06, 0.25] s/m of biologically plausible signal propagation (squares between vertical lines in Figure 2). The latter were reported in the literature using histological axonal bundles from the human brains (Caminiti et al., 2013). We further clarified the reliability of the model fitting via cross-validated model fitting (Jung et al., 2023). As a result, the selected parameter points (squares in Figure 2C–E) were stable across different subject configurations by random sampling (Supporting Information Figure S4).

The landscape of the group difference shows that the network modularity of PD patients is mostly higher than that of controls (Figure 2A). In contrast, the network efficiency of the patients is lower than that of the controls (Figure 2B).

The landscape of the correlations between the network modularity and the disease severity mostly shows significant positive correlations leading to one optimal parameter point of the strongest correlation (Figure 2C). On the other hand, the network efficiency of the simulated FC alters remarkably by varying the model parameters on the landscape leading to significant positive and negative correlations with disease severity (Figure 2D–E). With these landscape patterns, there are two optimal parameter points for the positive and negative correlations, respectively, and switching the optimal parameters from small to large delays impacts the brain dynamics leading to opposite tendencies of the correlations (Figure 2D–E). Therefore, we inferred that the simulated functional networks of patients may comparatively change when model parameters are varied, and there exist parameter domains of significant group difference in the network properties between the patients and controls (Figure 2A–B) as well as significant correlation between the network properties and disease severity (Figure 2C–E), where we can find optimal parameter points of the respective model fitting.

### Group Difference of Network Properties

**Figure 3.**
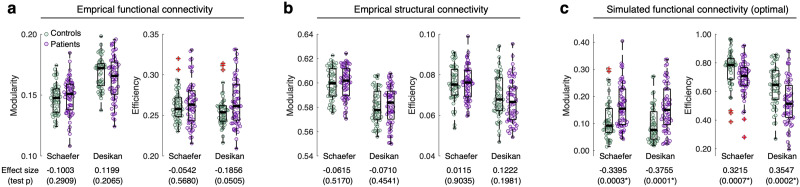
Group difference between healthy controls (HC, *n* = 51) and patients with PD (*n* = 60) for two brain parcellations and several comparison conditions. (A–C) The group differences of the network modularity and efficiency between HC and PD for (A) empirical FC, (B) empirical SC, and (C) optimal simulated FC. The empty circles in the plots correspond to individual subjects. The brain parcellations are indicated in the plots, and the values under the plots are the effect sizes of the group difference (positive for HC > PD and negative for PD > HC) and their statistics (*p* values of the Wilcoxon rank-sum two-tail test). The *p* values with asterisks indicate significant results (*p* < 0.05). The middle thick lines in the interquartile boxes indicate the medians of distributions, and the red crosses are the outliers.

We also calculated the network properties of the empirical connectomes, that are FC and SC, and compared the network properties of healthy controls with those of patients. We found no significant group differences in the empirical data (Figure 3A–B). In contrast, the network properties of the simulated FCs obtained for the optimal model parameters (squares in Figure 2A–B) show significantly different distributions between the controls and the patients. In the literature on a meta-analysis on empirical SC, the network modularity of patients was found to be higher, and the efficiency was found to be lower than those of healthy controls (Zuo et al., 2022). In the cross-sectional clinical data considered in this study, the network properties of the empirical FC and SC do not exhibit statistically significant differences between PD patients and healthy controls (Figure 3A–B). Nevertheless, we can still see weak but consistent tendencies for the used empirical SC in agreement with the literature (Zuo et al., 2022), where the modularity tends to be larger and the efficiency tends to be smaller for PD patients than for the healthy controls (Figure 3B). The differences in network properties of the empirical FC (Figure 3A) also show the same tendencies, which is in agreement with the literature (Kim et al., 2017; Nieuwhof & Helmich, 2017), except for the network modularity for the Schaefer atlas (Figure 3A).

Even though the network properties of the empirical data showed nonsignificant group differences, the network properties of the simulated FCs obtained for the optimal model parameters (squares in Figure 2A–B) show significantly different distributions between the controls and the patients. The modularity of patients is higher, and the efficiency is lower than those of healthy controls for both parcellations (Figure 3C), as reported in the literature on empirical SC (Zuo et al., 2022). Based on these findings, we conclude that the modeling results are different for healthy controls and PD patients. Consequently, the modularity and global efficiency of simulated whole-brain functional networks can be engaged in the relationship with clinical variables of PD, which we consider in detail below.

### Simulated Network Properties Reflect the Severity of the Disease

**Figure 4.**
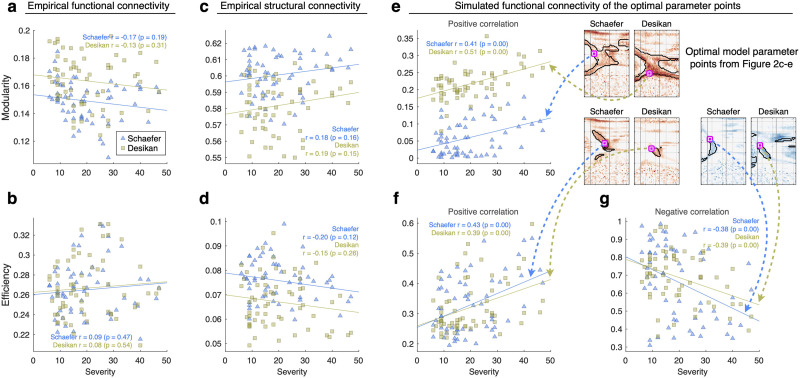
Correlation between severity of the disease of individual PD patients as given by the UPDRS (UPDRS III medication On, horizontal axes) and modularity and efficiency network properties (vertical axes) of empirical and simulated brain connectomes for (A–B) empirical FC, (C–D) empirical SC, and (E–G) simulated FC calculated for the optimal parameters of maximal correlations indicated by the dashed arrows from the optimal model parameter points from Figure 2C–E. The depicting triangles and squares in the plots denote that the two considered brain parcellations correspond to individual PD patients. The used brain parcellations, calculated Pearson’s correlation coefficient, and its statistical test (*p* value) are indicated in the legends.

We calculated Pearson’s correlation coefficients between the network properties (modularity and efficiency) and the disease severity (UPDRS III On). The network properties of the empirical FC and SC do not show significant correlation with disease severity (Figure 4A–D, Table 2). On the other hand, it is possible to find domains in the space of model parameters, where the network properties of the simulated FC generated by the model with these parameters show significant correlations with the disease severity (encircled regions in Figure 2C–E). The largest correlations between the network properties of the simulated FC and disease severity can be obtained for the group-level optimal parameter points (the magenta-white squares in Figure 2C–E), which are illustrated in Figure 4E–G (cf. Figure 4A–D for empirical data). Furthermore, we applied a cross-validated model fitting and found that optimal models derived from different subject subsamplings also showed significant correlations for unseen patients (Supporting Information Figure S4). Network modularity shows significant positive correlations with the disease severity (Figure 2C), but not significant negative correlations (Supporting Information Figure S5). Interestingly, the network efficiency of the simulated FC clearly shows two opposite tendencies in relation to the disease severity as reflected by positive and negative significant correlations with the disease severity (Figure 4F–G), which was observed for different parameter domains and, thus, for different optimal parameter points (Figure 2D–E). This contrasts with the network modularity that exhibits significant positive correlation only (Figures 2C and 4E). In the network topology of whole-brain connectivity (Rubinov & Sporns, 2010), high modularity corresponds to the presence of segregated (weakly interacting) network modules of densely interconnected nodes (brain regions) within modules. On the other hand, high efficiency is related to strong connections also between modules and can be interpreted as a measure of network integration.

**Table 2.** Correlation between empirical network properties and clinical variables (60 patients)

**Empirical FC**	**Age**	**Onset age**	**Disease duration**	**UPDRS III Off**	**UPDRS III On**	**UPDRS III Off − UPDRS III On**
Modularity (Schaefer)	−0.047	−0.143	0.166	−0.144	−0.172	0.050
Modularity (Desikan-Killiany)	−0.346*	−0.293*	−0.094	−0.072	−0.133	0.090
Efficiency (Schaefer)	0.287*	0.332*	−0.078	−0.023	0.095	−0.162
Efficiency (Desikan-Killiany)	0.358**	0.377**	−0.032	−0.014	0.080	−0.129
**Empirical SC**	**Age**	**Onset age**	**Disease duration**	**UPDRS III Off**	**UPDRS III On**	**UPDRS III Off − UPDRS III On**
Modularity (Schaefer)	0.230*	0.063	0.291*	0.159	0.183	−0.046
Modularity (Desikan-Killiany)	0.246	0.068	0.310*	0.170	0.186	−0.036
Efficiency (Schaefer)	−0.209	−0.054	−0.270*	−0.219	−0.204	−0.003
Efficiency (Desikan-Killiany)	−0.073	0.018	−0.158	−0.206	−0.149	−0.064

In the relationship, values are Pearson’s correlation coefficients (*n* = 60) denoted by asterisks (*p < 0.05 and **p < 0.005) as significant relationship.

In summary, using the parameter landscapes of the simulated network properties for individual subjects, we calculated the group-level statistical maps of the relationships (correlation) between the simulated brain networks and clinical measures (disease severity), where the model parameters and correlation values were controlled for the false discovery rate and conditioned on sufficiently large and noise-resistant intersubject variability. By doing so, we obtained parameter regions with significant correlations between network properties of the simulated FC and clinical measures. As a result, we arrived at the assertion that simulated brain networks with appropriately selected optimal model parameters reflect the severity of motor impairment in PD patients.

### Variability of Simulated Brain Networks Across Model Parameters

**Figure 5.**
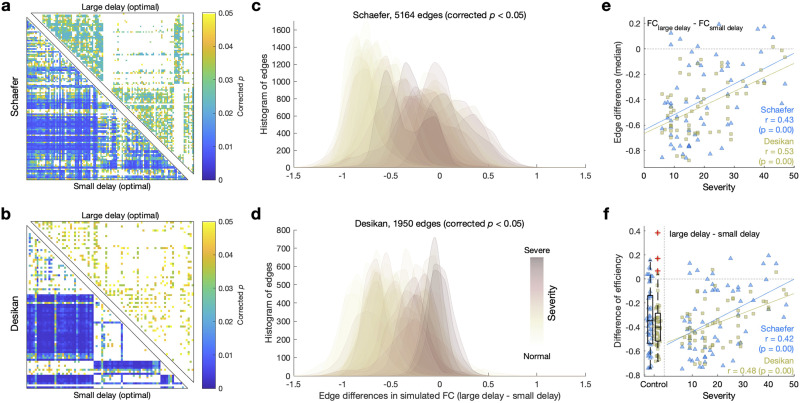
Correlations between disease severity as given by the unified PD rating scales (UPDRS III, medication On) and simulated FC. The latter was simulated for the optimal model parameters of the strongest positive and negative correlations between the disease severity and network efficiency of simulated FC obtained for large and small optimal delays, respectively (Figure 2D–E, square marks). (A–B) Results of statistical tests (*p* values corrected by the Benjamini-Hochberg false discovery rate) of Pearson’s correlation (across patients) between the disease severity and the edges of the simulated FC for the optimal model parameters of small (lower triangles) and large (upper triangles) delays for each parcellation indicated at the left. (C–D) Histograms of the differences of significant FC edges from lower triangles of the corrected *p* matrices (A–B) between the large and small optimal delays of each patient and parcellation scheme. The color shading of the histograms indicates the severity of the disease of corresponding patients. (E–F) Scatterplots of the relationships between the disease severity and the differences between the values for the large and small delays of (E) the medians of the histograms in (C–D) and (F) the network efficiency. The depicting triangles and squares in the plots denote the two considered brain parcellations correspond to individual PD patients. The amount of correlation of the depicted relationships are indicated in the plots together with the results of its statistical tests (*p* values) of Pearson’s correlation for both considered parcellation schemes. In the scatterplot (F, left side), the boxplots depict the distributions of the respective values of the efficiency differences (vertical axes) for 51 healthy controls, where the middle lines in the interquartile boxes indicate the medians of distributions, and the red crosses are outliers. Both distributions were normally distributed according to the Kolmogorov-Smirnov normal distribution test and significantly different from zero (one-sample, two-tail *t* test).

To investigate the changes in the simulated brain network topology as the model parameters vary, we in detail analyzed the connections (network edges) of the simulated FC across the patients. We focused on the intriguing case of the network efficiency of the simulated FC, identifying two different optimal parameter points corresponding to the strongest positive (Figures 2D and 4F) and negative (Figures 2E and 4G) correlations between the network efficiency and the disease severity. These two parameter points in particular differ from each other by the values of delay such that the positive and negative correlations are exhibited for larger and smaller delays, respectively (Figure 2D–E), which also result in different simulated FCs. In the case of small delay, many interhemispheric connections significantly negatively correlate with the disease severity (lower triangles in Figure 5A–B). On the other hand, in the case of large delay, the situation is reversed, where many intrahemispheric connections significantly positively correlate with the disease severity instead (upper triangles in Figure 5A–B).

The case of the negative correlation between the network efficiency and disease severity might be related to the results for empirical data, where the efficiency of SC was shown in the literature to decrease in PD (Zuo et al., 2022). This can hardly be recognized for our empirical data used for the modeling, neither for the efficiency differences (Figure 3A–B) nor for its correlations with the disease severity (Figures 4B and 4D). Our research thus suggests that we can identify brain network alterations with simulated FC using cross-sectional data, which is not possible with empirical SC and FC. Furthermore, the case of positive correlations was revealed by the model as a possible hidden state that can be used for comparison.

Based on the above results showing the opposite tendencies of the correlation, we therefore opted for the significant FC edges obtained for the optimal parameters with small delays (negative correlation) as edges of the interest (nonwhite edges in lower triangles in Figure 5A–B) for further analysis. Thereafter, we subtracted the selected FC edges of small delay from the corresponding FC edges of large delay for every subject (FC of large delay − FC of small delay) and related these edge differences to the disease severity of a given subject.

Interestingly, the two model regimes are different from each other (large edge differences) for the patients with less severe motor impairment, while the edge differences approach the origin when the severity increases (Figure 5C–D). Accordingly, the medians of the edge differences exhibit the same behavior and significantly correlate with the disease severity (Figure 5E). Similar behavior is also observed for the corresponding network efficiencies, where their difference also significantly correlates with the disease severity and approaches zero when the disease severity increases (Figure 5F). We may additionally compare the discussed efficiency differences with those for the healthy controls calculated for the same optimal parameter values as before (leftmost boxplots in Figure 5F). The efficiency differences of healthy controls significantly and strongly deviate from zero, which could be interpreted as an extent of the efficiency variability across a model parameter space for the limiting case of zero disease severity. Our results demonstrate that the simulated brain networks of patients with severe symptoms show little variability in the network efficiency across a model parameter space in contrast to the healthy condition. This information could be used to evaluate such a variability and diagnose the disease severity.

### Relations Between Network Properties and Disease Duration

In the previous sections, we presented the results for the group differences and relationships between the network measures and the disease severity. In this section, we focus on the disease duration instead. The network properties of empirical FC do not show a significant correlation with the disease duration, but those of the empirical SC are significantly correlated with the disease duration (Table 2). To evaluate the impact of the disease duration on the network properties of simulated FC, we applied the behavioral network-based model fitting to the disease duration instead of the disease severity considered above (Supporting Information Figure S6A–B).

**Figure 6.**
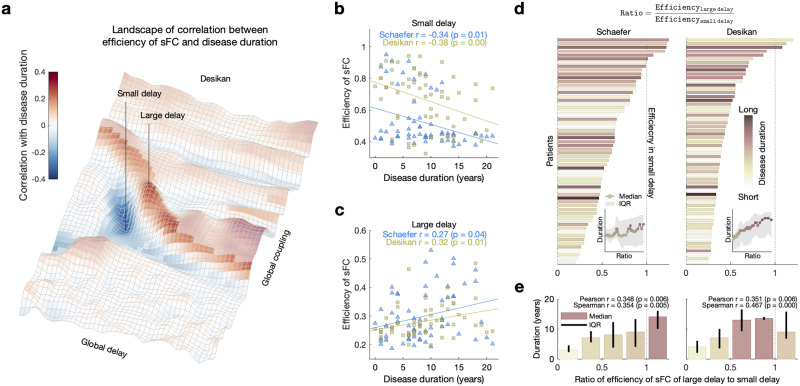
Relationships between network efficiency of simulated FC and disease duration. (A) Parameter landscape of Pearson’s correlation coefficients between the simulated network efficiency and the disease duration for the Desikan-Killiany (Desikan) atlas. The vertical lines with “small delay” and “large delay” indicate optimal parameter points for negative and positive correlations, respectively. (B–C) Scatterplots for (B) negative and (C) positive correlations between disease duration and network efficiency of the simulated FC at the optimal parameter points with small and large delay, respectively. The lines are linear fitting between simulated network efficiency and the disease duration. The depicting triangles and squares in the plots denote the two considered brain parcellations and correspond to individual PD patients. The amount of correlation of the depicted relationships are indicated in the plots together with results of statistical tests (*p* values) for both considered parcellation schemes. (D) Ratio of the optimal simulated network efficiency of large delay to that of small delay for individual patients (vertical axes) sorted according to the ratio of the network efficiency indicated by the horizontal bars with color depicting the disease durations of the corresponding patients. The inserts show medians of the disease duration corresponding to the moving average along the patients in ascending order of the efficiency ratio. The gray shadow indicates interquartile ranges (IQR) of the disease duration. (E) Barplots of the median values of the efficiency ratio with error bars indicating IQR of ratios in five intervals splitting the range from 0 to 1.25. The amount of correlation of the depicted relationships between the efficiency ratio and the disease duration is denoted in the plots together with results of statistical tests (*p* values) of the Pearson’s correlation and the Spearman’s correlation, respectively.

The network modularity of simulated FC demonstrates significant positive correlations with the disease duration for the optimal parameter points, which is consistent with the case of the empirical SC (Table 2, Supporting Information Figure S6A). Interestingly, for the network efficiency, we found parameter regimes of both significant negative and positive correlations with the disease duration (Figure 6A–C, Supporting Information Figure S6B) as for the relationship with the disease severity (Figure 2D–E). This is in spite of a weak negative correlation between the disease duration and severity (Table 1).

As in Figure 5, in this section, we investigated how the simulated FC network changes when the model varies between two optimal parameter points. Since the modularity has only one such point of positive correlation with the disease duration, we considered the network efficiency, where two optimal parameter points exist of positive correlation (larger delay) and negative correlation (smaller delay); see Figure 6A. Then, we calculated a ratio of the optimal efficiency value for large delay to that for small delay for every patient (Figure 6D). We observed that the network efficiency is larger for the optimal parameter point of negative correlation (ratio <1) for most patients, and it decays for a longer course of the disease. This resulted in a smaller difference between the two optimal efficiency values for a longer disease history, such that the efficiency ratio grows toward 1 and slightly beyond with significant positive correlation between the ratio and the disease duration (Figure 6E). Therefore, for the patients with longer disease duration, the network efficiency is expected to change little between the two optimal parameter points, which might be considered as a useful information for disease diagnosis. In summary, with the considered modeling approach using cross-sectional clinical data, we showed that simulated network properties relate to the progression of PD throughout the well-pronounced correlation with the disease severity and duration.

## DISCUSSION

The aim of the current study was to demonstrate the relationship between simulated brain network properties and clinical variables while considering the progression of PD, such as severity of motor impairment and disease duration. The reported results indicate that functional segregation and integration of simulated brain networks can significantly correlate with disease severity (UPDRS III). In addition, the simulated network properties provide higher effect sizes for the group difference between healthy controls and PD patients than those of empirical networks. Remarkably, alterations in efficiencies of simulated brain networks derived from the models with distinct optimal parameters evidently reflect the clinical measures (the severity and duration of the disease). As a potential approach, the behavioral network-based model fitting allows us to explore simulated brain networks that reflect the progression of the disease. Consequently, we suggest a way to generate and utilize simulated human connectomes for investigation of behavioral or clinical measures and disease onset and progression.

The results of the current study indicate that simulated data of whole-brain dynamical models can provide features of brain networks with a great potential for enhancing group differences between healthy controls and patients with PD. Furthermore, the simulated brain networks clearly reflect clinical scores such as disease severity and duration. Based on the modeling results, the simulated brain networks outperform the empirical networks in regard to group differences and correlations with clinical variables. In contrast, empirical FC did not exhibit such clear relationships with the modeling, and empirical SC only showed significant correlation with disease duration (Table 2). Thus, the whole-brain modeling is essential to reveal relationships between brain networks and clinical measures. In contrast to using empirical brain networks, the data-driven modeling approach (Popovych et al., 2019) allows us to explore model parameters and search for the most effective models that simulate brain dynamics at the best correspondence to the posed research questions.

The simulation results for the efficiency of the functional networks are more involved with disease severity than those for the modularity, because the former show both negative and positive correlations (Figure 4F–G) with disease severity. In addition, efficiency exhibits regimes that are associated with alteration in the interhemispheric connections in simulated brain networks. In other words, interhemispheric connections in the brain of the patients with severe motor impairment are not adequately contributing to whole-brain dynamics. This interpretation is supported by the literature addressing that the interhemispheric connections are related with the functional integration (Gotts et al., 2013). Additionally, decreased interhemispheric connections in PD were also observed in the empirical SC in the current study (Supporting Information Figure S7). This is consistent with the empirical result of decreased resting-state interhemispheric connections in PD (Luo et al., 2015).

The severity of motor impairment of PD is expected to increase over time (Holden et al., 2018) because of the progressive disease. Although we observe a weak correlation between the severity and duration of the disease in these cross-sectional clinical scores (Table 1), we interpret our modeling results (Figures 5 and 6) as the impact of the disease progression on the simulated network efficiency, which reflects the disease severity and duration. In addition, the positive correlation between the difference of UPDRS III scores (Off − On) and the disease duration (Supporting Information Figure S1B) allows us to interpret the effect of medication as being related to the disease duration (Manza et al., 2017). With this, we infer that the alteration of the simulated network efficiency can intermediate the relationship of the disease duration with the effect of medication. Therefore, the simulation outcomes of the dynamical models can be used to integrate the relationships among the disease progression (disease severity and duration) and the effect of medication via interrelating them with topological properties of simulated brain networks (see Figure 1C). In summary, our results support the claim that the whole-brain dynamical modeling can provide a potential way for understanding the interrelations between the properties of brain networks and PD progression and severity.

One of the main advances of the current novel approach is that the calculated parameter landscapes of simulated network properties provide statistical maps and bases for the behavioral model fitting. Subsequently, significant regimes in a landscape can be selected for the best correspondence to target variables and research questions. This analysis has some analogy with statistical brain mapping in neuroimaging research related with behavioral tasks (Friston et al., 1994), clinical tests (Thompson et al., 2004), genetic measures (Hawrylycz et al., 2012), and so forth. Therefore, we can also apply this approach to the parameter landscapes (mapping) of the simulated brain dynamics that can be related to behavioral and clinical measures as we demonstrated in this study.

The modeling and validation methods employed in this study rely on empirical SC and clinical variables, instead of empirical FC as a target variable for model fitting. In our previous study, we used the empirical FC as an objective for model fitting, that is Pearson’s correlation between empirical and simulated FC, and obtained a similarity from 0.2 to 0.4 (Jung et al., 2023). The optimal models in this study were optimized by behavioral model fitting to the disease severity and duration and showed a slightly lower similarity between the empirical and simulated FC from 0.05 to 0.35 (Supporting Information Figure S8). Nevertheless, we obtained an enhanced and robust relationship between the clinical scores as given by disease severity and duration and the network properties of simulated FC and their alterations, which cannot be observed from the empirical cross-sectional clinical data used in this study. This may have a few advantages including a possible positive influence on reliability of simulation results. Indeed, empirical FC and SC have different test-retest reliabilities, where the empirical resting-state FC showed reliability in the range from 0.3 to 0.6 (multisites with various scan times; Andellini et al., 2015; Domhof et al., 2022; Zuo et al., 2013) in the intraclass correlation coefficient (ICC). On the other hand, ICC values of empirical SC were found between 0.7 and 0.8 (intrasite, intersite, and multisites; Owen, Ziv, et al., 2013; Zhao et al., 2015). Accordingly, network properties of empirical SC also showed a higher reliability than that of empirical resting-state FC (Nakuci et al., 2023). With such different reliabilities, the current modeling approach could also show varied results when we use different study conditions. Therefore, we applied a cross-validated model fitting approach (Jung et al., 2023) to the landscapes of correlations between simulated network efficiency and disease severity. As a result, it shows stable optimal model parameter points across different patient configurations by random sampling (Supporting Information Figure S4). Therefore, the suggested behavioral network-based model fitting may show consistent outcomes when we include unseen subjects into the analysis.

The current whole-brain model considers the excitatory and inhibitory populations in each brain region and (local and global) interactions among them, which represent neurophysiological activities in coupled cortical columns (Jansen & Rit, 1995). However, the current model is still relatively simple compared with the network of such a complex human brain. Thus, it is necessary to investigate individualized whole-brain models with varied local parameters instead of fixed values such as excitatory-inhibitory balances in the future study. For generalized modeling, the current approach needs to be evaluated by including multisite data.

The considered global network measures are not able to reflect regional variations in the brain network such as, for example, local clustering coefficient or local efficiency. The global network properties provide one representative value per brain network, which was utilized in this study for the whole-brain model fitting. Other network measures might be interesting to consider in this context, including the clustering coefficient, characteristic path length, network strength and density, as well as small-worldness and local efficiency in order to compare the modeling results with the changes induced by PD to these measures reported for empirical data (Zuo et al., 2022).

Investigating alterations of the brain connectome is essential for understanding the progression of neurodegenerative diseases. Accordingly, our findings can be utilized for future research that can show the impact of the disease duration on the whole-brain dynamics. Furthermore, including prodromal subjects and longitudinal data will provide a way to validate the current approach as progressive markers. Therefore, future investigations about the impact of the progression of PD on disease symptoms and brain networks will use longitudinal data that consist of healthy, prodromal, and diseased subjects from multiclinical sites. For an advanced approach, we can consider individualized whole-brain models with varied local parameters using high-dimensional parameter optimization (Sip et al., 2023) and its applications, for instance, a computational biomarker for individual clinical scores. As a consequence, the modeling outcome can be used for objective evaluations of these clinical indices.

## MATERIALS AND METHODS

### Participants

Multimodal brain MRI data were acquired in 111 human subjects including 51 healthy subjects (21 females; age range: 41–78 years) and 60 patients (17 females; age range: 44–80 years) with PD. The age of symptom onset and disease duration were acquired in PD patients. Furthermore, the UPDRS (Goetz et al., 2008) was assessed by an expert neurologist for each patient in a condition under regular medication (UPDRS-On) and after at least 12 hr of withdrawal of all dopaminergic drugs (UPDRS-Off). All healthy controls had no history of any neurologic or psychiatric disease and had no abnormalities on cranial MRI. The study was approved by the local ethics committee and performed in accordance with the Declaration of Helsinki. Written informed consent was obtained prior to study inclusion.

### MRI Protocols and Processing

A 3T scanner (Siemens Trio) was used for T1-weighted MRI (T1w; voxel size = 1.0 × 1.0 × 1.1 mm^3^), diffusion-weighted MRI (dwMRI; B = 1,000 s/mm^2^ with 64 directions; voxel size = 2.4 × 2.4 × 2.4 mm^3^) with a nonweighted (B_0_) image, and resting-state functional MRI (rs-fMRI; TR = 2.21 s; 300 volumes during 663 s; voxel size = 3.125 × 3.125 × 3.565 mm^3^). MRI processing was performed using a pipeline that included structural and functional modules. The structural module performed preprocessing for T1w (bias-field correction, alignment of anterior-posterior commissures, brain tissue segmentation, reconstruction of gray-white matter boundary and pial surface) and dwMRI (removing the Gibbs ringing effect; correction of bias-field, head-motion, and eddy distortion). Subsequently, the whole-brain tractography (WBT) with 10 million streamlines was calculated based on the estimated fiber orientation distributions of white matter using spherical deconvolution (Tournier et al., 2007, 2019). The functional module processed rs-fMRI (correction of slice time and head motion; reslicing in a 2-mm isocubic voxel space; intensity normalization; detrending; nuisance regression with regressors of white matter, cerebrospinal fluid, the entire brain, and the head motion). Two brain atlases were used for cortical parcellation based on the Schaefer (Schaefer et al., 2018) atlas with 100 parcels and Desikan-Killiany (Desikan et al., 2006) atlas with 68 parcels. The following 14 subcortical regions were also added into each atlas: thalamus, caudate, putamen, pallidum, hippocampus, amygdala, and accumbens from each hemisphere segmented based on the automated labeling technique (Fischl et al., 2002). The pipeline counted streamlines in WBT connecting any two brain regions, which shaped SC, and calculated average path length of the given streamline bundles between the regions, which was used to calculate model delays of neural signal propagation between brain regions. It also extracted the mean BOLD signals of each brain region from the processed rs-fMRI and calculated Pearson’s correlation coefficients between the BOLD signals of any two brain regions, which constitutes FC. More details of the MRI processing and tracking parameters can be found elsewhere (Jung et al., 2023).

### Empirical Network Properties

For the empirical functional network properties, we took the absolute values of edges in empirical FC. By doing this, we calculated the network modularity and global efficiency for the same network for FC and SC, and the brain connectivity toolbox was used to calculate these network measures (Rubinov & Sporns, 2010). For the empirical structural network properties, streamline counts of edges in empirical SC were divided by the maximal number of streamlines (self-connections were excluded) and used for the network properties as well.

### Neural Mass Model of Two Neural Populations

The postsynaptic potentials (PSPs) were simulated through interactions between excitatory and inhibitory neural populations in each brain region based on the Jansen-Rit model type (Jansen & Rit, 1995; Lopes da Silva et al., 1974) as a convolution-based neural mass model (Moran et al., 2013) transforming the average of presynaptic firing density into the average PSP between excitatory neural populations in brain regions. Simulated PSP signals were obtained by using the following differential equations:

$${\dot{y}}_{n,e}\left(t\right)=z_{n,e}\left(t\right),$$ (1)

$${\dot{y}}_{n,i}\left(t\right)=z_{n,i}\left(t\right),$$ (2)

$${\dot{z}}_{n,e}\left(t\right)=P_{n,e}\left(t\right)-2aRz_{n,e}\left(t\right)-a^{2}R^{2}y_{n,e}\left(t\right)+\eta_{n,e},$$ (3)

$${\dot{z}}_{n,i}\left(t\right)=P_{n,i}\left(t\right)-2bRz_{n,i}\left(t\right)-b^{2}R^{2}y_{n,i}\left(t\right)+\eta_{n,i},$$ (4)

n=1,2,…,N.

Here, *y*_*n*,*e*_, *y*_*n*,*i*_, *z*_*n*,*e*_, and *z*_*n*,*i*_ are excitatory and inhibitory PSPs, and excitatory and inhibitory postsynaptic current, respectively, in the *n*^th^ brain region out of *N* brain regions depending on parcellation granularity. The subscripts *e* and *i* of the variables indicate excitatory and inhibitory populations, respectively. Parameters *a* and *b* are the reciprocal of the time constants of the PSP kernel for the excitatory and inhibitory populations. A random uniform distribution was used for independent noise *η*, and *R* scaled the spectral power distribution of the PSP signals.

The models of different brain regions were coupled through the excitatory populations, where the empirical structural connectome was employed to calculate the coupling strengths and delays forming a network backbone of the whole-brain model. The intra- and interregion coupling is included in the terms *P*_*n*,*e*_ and *P*_*n*,*i*_, serving as inputs to the excitatory and inhibitory populations in region *n*, respectively, and has the following form:

$$P_{n,e}\left(t\right)=AaR^{2}\sigma_{e}\left(\frac{C}{N}\sum_{m\neq n}^{N}C_{nm}y_{m,e}\left(t-\tau_{nm}\right)-C_{ei}y_{n,i}\left(t\right)\right),$$ (5)

$$P_{n,i}\left(t\right)=BbR^{2}\sigma_{i}\left(C_{ie}y_{n,e}\left(t\right)\right).$$ (6)

Parameters *A* and *B* are the maximal amplitudes of the excitatory and inhibitory PSP kernels. The interregional coupling strength and its delay from region *m* to region *n* (*C*_*nm*_ and *τ*_*nm*_) can be estimated using the following empirical structural connectome:

$$C_{nm}=\frac{w_{nm}}{\langle W\rangle },$$ (7)

$$\tau_{nm}=\tau L_{nm},$$ (8)

where *w*_*nm*_ and *L*_*nm*_ are the number and the average path length of streamlines, respectively, between region *m* and region *n*, where the former was normalized by the averaged number of streamlines of the structural connectome 〈*W*〉. Parameters of the global coupling *C* (arbitrary unit) and global delay *τ* (s/m) were used to scale couplings and delays throughout the whole-brain network. The coupling weights *C*_*ie*_ and *C*_*ei*_ balance the interactions from excitatory to inhibitory populations and vice versa. The inter- and intraregion coupling involved an averaged firing density calculated by the following sigmoid functions:

$$\sigma_{e}\left(v\right)=\frac{F_{e}}{1-e^{r\left(v_{0}-v\right)}}\;\text{and}\;\sigma_{i}\left(v\right)=\frac{F_{i}}{1-e^{r\left(v_{0}-v\right)}}$$ (9)

Here, *r* is a slope of the sigmoid function, and *v*_0_ is a half of the maximal membrane potentials. *F*_*e*_ and *F*_*i*_ are the maximal firing densities of the excitatory and inhibitory populations. The simulated excitatory PSP signals were applied to the neurovascular coupling and the hemodynamic function as described by the Balloon-Windkessel model (Buxton et al., 1998; Friston et al., 2000), which converted the simulated electrical neural activity to simulated BOLD signals. More details of the whole-brain model and its parameter values can be found elsewhere (Havlicek et al., 2015; Jansen & Rit, 1995; Jung et al., 2023).

### Implementation of Simulation

The whole-brain model (Equations 1–9) was simulated by a custom-made program written in C++, with an integration step of 2 ms during 720 s, where the first 57 s were discarded as a transient. The remaining 663 s (the same as the length of empirical rs-fMRI) were used for analysis. The simulation was carried out on the high-performance computing cluster (Thörnig, 2021). The global coupling and global delay were varied as free model parameters on a dense grid of 64 values of global couplings [0, 63] and 43 values of global delays [0, 0.42] leading to 2,752 model runs for each subject and parcellation.

### Network-Based Behavioral Model Fitting and Parameter Selection

Simulated BOLD signals were used to calculate the simulated FC by the pairwise Pearson’s correlation between the simulated BOLD signals of the brain regions. For the *behavioral network-based model fitting*, the graph-theoretical network properties (modularity and efficiency; Rubinov & Sporns, 2010) of the simulated FC matrices were obtained for each model parameter point. To calculate the network properties, we took absolute values of the edges in the simulated FC matrices as for the empirical FC and delineated a parameter landscape (64 × 43 grid of model parameter points) using the network properties of the 2,752 simulated FC. In the end, each subject had two parameter landscapes of network modularity and efficiency for every parcellation. With this, group-level analyses and statistical tests were performed for statistical mapping on the landscapes across subjects.

Under each condition (network property and atlas), two masks were used to select parameter domains out of the entire model parameter space for further analyses (Figure 2, right). First, we only considered parameter points with statistically significant relationships between the simulated network properties and empirical data, for example, Pearson’s correlation coefficients between the network properties and the severity of the disease (Supporting Information Figure S2A). Second, we restricted our analyses to parameter domains with sufficiently high intersubject variability in the network properties. In this context, the standard deviation of the considered network properties of simulated FC was calculated across all subjects for every parameter point of the model parameter plane, and the parameter domains with large variability (>75% across all parameter points) were encircled and used to mask the model parameters (Supporting Information Figure S2B). Considering such a parameter mask can positively contribute to the investigation of the interindividual differences and resistance against noise, see Supporting Information Figure S3, where the resistance against noise is illustrated for network efficiency. The parameter points from the overlap of the two masks (Figure 2, right and Supporting Information Figure S1C) were used for the model fitting and for finding optimal parameter points.

Consequently, we can search for the optimal model parameters corresponding to the strongest correlation between the network properties and clinical scores (severity of the disease and disease duration) and the largest effect size of the group difference between healthy controls and PD patients. To see how the optimal parameter points are stable across different groups of sampled patients, we performed a stratified threefold cross-validation for the behavioral network-based model fitting (200 iterations). Subsequently, Pearson’s correlation coefficients between disease severity and the considered network properties were calculated during training and testing steps.

### Statistical Analysis

Effect sizes of the group difference between healthy subjects and patients were calculated by the Rosenthal formula (Rosenthal et al., 1994), which uses a *z*-statistic utilized to compute the *p* value of the nonparametric Wilcoxon rank-sum two-tail test (*n* = 111). The statistics of the Pearson’s correlation coefficient between network properties and clinical or demographic variables were calculated for testing the hypothesis that there is no linear relationship (null hypothesis) between observations (*n* = 60). The Benjamini-Hochberg procedure (Benjamini & Hochberg, 1995) was applied for controlling the false discovery rate (FDR) of a family of hypothesis tests from the edge-wise statistics performed multiple times using Pearson’s correlation (corrected *p* values). The random-field theory (Ashburner et al., 2003; Worsley et al., 1996) for multiple tests was applied to statistical landscapes, and significant areas were thresholded (*Z* > 3.82 as corrected *p* < 0.05). The Kolmogorov-Smirnov test (Massey, 1951) was used for normality testing of differences in the efficiency of healthy controls (*n* = 51), and a one-sample, two-tail *t* test was applied for testing the null hypothesis (no difference from zero) of the distribution. Statistical tests with *p* < 0.05 were considered to confirm the significance of the results. The Benjamini-Hochberg FDR procedure was employed in the Mass Univariate ERP Toolbox (Groppe et al., 2011). All statistical tests were performed in MATLAB (R2020b; MathWorks).

## ACKNOWLEDGMENTS

The primary contact for the UKD-PD team is Professor Doctor Julian Caspers (Julian.Caspers@med.uni-duesseldorf.de). The authors gratefully acknowledge the computing time granted through JARA (Jülich Aachen Research Alliance) on the supercomputer JURECA (Jülich Research on Exascale Cluster Architectures) at Forschungszentrum Jülich. This work was supported by the Portfolio Theme Supercomputing and Modeling for the Human Brain by the Helmholtz association, the Human Brain Project and the European Union’s Horizon 2020 Research and Innovation Programme under Grant Agreements 785907 (HBP SGA2), 945539 (HBP SGA3), and 826421 (VirtualBrainCloud). Open-access publication was funded by the Deutsche Forschungsgemeinschaft - 491111487. The funders had no role in the study design, data collection and analysis, decision to publish, or preparation of the manuscript.

## SUPPORTING INFORMATION

Supporting information for this article is available at https://doi.org/10.1162/netn_a_00406.

## AUTHOR CONTRIBUTIONS

Kyesam Jung: Conceptualization; Data curation; Formal analysis; Investigation; Methodology; Resources; Software; Validation; Visualization; Writing – original draft; Writing – review & editing. Simon B. Eickhoff: Conceptualization; Funding acquisition; Project administration; Resources; Writing – review & editing. Julian Caspers: Data curation; Writing – review & editing. UKD-PD team: Data curation. Oleksandr V. Popovych: Conceptualization; Funding acquisition; Methodology; Project administration; Resources; Software; Supervision; Validation; Writing – review & editing.

## ADDITIONAL INFORMATION

UKD-PD team (Universitätsklinikum Düsseldorf - Parkinson’s disease)

Julian Caspers^3^, Christian Mathys^3,4,5^, Martin Südmeyer^6,7^, Felix Hoffstaedter^1,2^, Christian J. Hartmann^6^, Christian Rubbert^3^, Alfons Schnitzler^6^, Bernd Turowski^3^

^4^Institute of Radiology and Neuroradiology, Evangelisches Krankenhaus Oldenburg, Universitätsmedizin Oldenburg, 26122 Oldenburg, Germany

^5^Research Center Neurosensory Science, Carl von Ossietzky Universität Oldenburg, 26129 Oldenburg, Germany

^6^Department of Neurology and Institute of Clinical Neuroscience and Medical Psychology, Medical Faculty and University Hospital Düsseldorf, Heinrich Heine University Düsseldorf, 40225 Düsseldorf, Germany

^7^Department of Neurology, Ernst-von-Bergmann Klinikum, 14467 Potsdam, Germany

## FUNDING INFORMATION

Simon B. Eickhoff, H2020 Future and Emerging Technologies (https://dx.doi.org/10.13039/100010664), Award ID: 785907. Simon B. Eickhoff, H2020 Future and Emerging Technologies (https://dx.doi.org/10.13039/100010664), Award ID: 945539. Simon B. Eickhoff, H2020 Health (https://dx.doi.org/10.13039/100010677), Award ID: 826421. Simon B. Eickhoff, Deutsche Forschungsgemeinschaft (https://dx.doi.org/10.13039/501100001659), Award ID: 491111487.

## DATA AND CODE AVAILABILITY

The clinical data used in this study are not immediately available for public sharing because the given informed consent of the patients did not include public sharing. The simulated data that support the findings of this study are available from the corresponding author upon a reasonable request. The brain connectivity toolbox is available here (https://sites.google.com/site/bctnet/). The containerized pipeline is publicly available (https://jugit.fz-juelich.de/inm7/public/vbc-mri-pipeline). The Mass Univariate ERP Toolbox is a publicly available software (https://openwetware.org/wiki/Mass_Univariate_ERP_Toolbox). The convolution-based neural mass model is also available from the first author upon a reasonable request.

## Supplementary Material



## TECHNICAL TERMS

Model fitting: Model fitting adjusts a computational model’s parameters to match empirical data. In this study, model fitting aligns simulated network properties with clinical scores.
Network modularity: Network modularity measures the division strength and segregation of brain networks into distinct modules, indicating dense intramodule and sparse intermodule connections.
Network global efficiency: Network global efficiency measures information exchange efficiency and overall integration, calculated as the average inverse shortest path lengths, indicating ease of communication in brain networks.
UPDRS III (Unified Parkinson’s Disease Rating Scale part III): UPDRS is an assessment of both motor and nonmotor symptoms associated with Parkinson’s disease. The part III is a clinician-scored monitored motor evaluation.
Whole-brain model: Whole-brain model simulates neural activity across the entire brain using computational models based on brain imaging data. It analyzes interactions between brain regions to study brain dynamics, linking behavioral or clinical measures.
Brain parcellation (brain atlas): Brain parcellation divides the brain cortex into distinct regions using brain atlases, aiding in the study of brain function, structure, connectivity, and network.
